# Supplementary material for: Content-rich biological network constructed by mining PubMed abstracts
Source: BMC Bioinformatics. 2004 Oct 8;5:147. doi: 10.1186/1471-2105-5-147 (PMC528731; doi:10.1186/1471-2105-5-147)
Supplement: Additional File 5 — The original Chilibot query results of the term "long-term potentiation (LTP)" and 22 other terms, limiting the latest references analyzed to the years 1990, 1995, 2000, and 2004. [file 1471-2105-5-147-S5.bz2 › chilibotAdditionalFile5/ltp2004/html/PI-3K_TAU.html]

 


 **PI-3K** and **TAU** 
  
Found 8 abstracts in PubMed,  **8 abstracts were retrieved and analyzed**.  


---

 Search Google  |
 PDF files only 
|  EDU domain only 

---

**Interactive relationship** (e.g. stimulation, inhibition, etc)

- A beta inactivates PI3 kinase  [ **PI-3K** ]  and activates TPK I GSK 3 beta, which in turn phosphorylates and inactivates both  **tau**  and pyruvate dehydrogenase PDH.  Ref: 9816911 Rinsho Byori, 1998
- Wortmannin, an inhibitor of PI 3 kinase  [ **PI-3K** ] , also activated TPK I GSK 3 beta, leading to an enhancement of  **tau**  phosphorylation and neuronal death in hippocampal culture.  Ref: 8742040 Neurosci Lett, 1996
- This effect is specific to profilin and gelsolin because other cytoskeletal proteins such as  **tau**  or actin do not affect PI 3 kinase  [ **PI-3K** ]  activity.  Ref: 8987988 Biochemistry, 1996

**Parallel relationship** (e.g. studied together, co-existance, homology, etc.)

- These results suggest that A beta 25 35 inhibition of PI 3 kinase  [ **PI-3K** ]  results in the activation of TPK I GSK 3 beta, the phosphorylation of  **tau** , and resultant neuronal death in rat hippocampal neurons.  Ref: 8742040 Neurosci Lett, 1996
- Here, we show that one of the CXCR2 ligand GROalpha KC can be a potent trigger for the ERK1 2 and PI 3 kinase  [ **PI-3K** ]  pathways, as well as  **tau**  hyperphosphorylation in the mouse primary cortical neurons.  Ref: 11777543 J Neuroimmunol, 2002
- In order to examine the effects of A beta treatment on intracellular signaling mechanism, we have investigated the role of phosphatidyl inositol 3 PI 3 kinase  [ **PI-3K** ]  in the phosphorylation of  **tau** .  Ref: 8742040 Neurosci Lett, 1996
- GROalpha KC, a chemokine receptor CXCR2 ligand, can be a potent trigger for neuronal ERK1 2 and PI 3 kinase  [ **PI-3K** ]  pathways and for  **tau**  hyperphosphorylation a role in Alzheimer s disease ? < ArticleTitle>.  Ref: 11777543 J Neuroimmunol, 2002
